# Clinical trials to estimate the efficacy of preventive interventions against malaria in paediatric populations: a methodological review

**DOI:** 10.1186/1475-2875-8-23

**Published:** 2009-02-10

**Authors:** Vasee S Moorthy, Zarifah Reed, Peter G Smith

**Affiliations:** 1 Nuffield Department of Clinical Medicine, John Radcliffe Hospital, Oxford, OX3 9DU, UK; 2 Initiative for Vaccine Research, World Health Organization, 20 Avenue Appia, 1211 Geneva 27, Switzerland; 3 London School of Hygiene and Tropical Medicine, London, WC1E 7HT, UK

## Abstract

**Background:**

Recent years have seen publication of a considerable number of clinical trials of preventive interventions against clinical malaria in children. There has been variability in the specification of end-points, case definitions, analysis methods and reporting and the relative lack of standardization complicates the ability to make comparative evaluations between trials.

**Methods:**

To prepare for a WHO consultation on design issues in malaria vaccine trials, controlled trials of preventive interventions against malaria in children in endemic countries were identified in which clinical malaria, or death, had been one of the main end-points. Trials were included that evaluated the impact of vaccines, insecticide-treated bed nets (ITN), intermittent presumptive or preventive therapy in infants (IPTi) or, in one instance, vitamin A supplementation. Methods that had been used in these trials were summarized and compared in order to identify issues that were directly relevant to the design of malaria vaccine trials.

**Results:**

29 controlled trials of preventive malaria interventions were identified, of which eight were vaccine trials. Vaccine trials that were designed to detect an effect on clinical malaria all reported the incidence rate of first episodes of clinical malaria as their primary endpoint. Only one trial of a preventive intervention (of ITN) was identified that was designed to detect an effect on severe malaria. A group of larger trials were designed to detect an effect of impregnated bed nets or curtains on all-cause mortality as the primary end-point. Key methodological and reporting differences between trials are noted in the text. Two issues have been identified that are of some concern. Firstly, the choice of primary endpoint is not stated in the reports of a number of the trials and, secondly, the relationship between pre-specified analysis plans and trial reports is rarely made clear.

**Conclusion:**

This article reports an investigation into the ways in which trial design and reporting could be improved and standardized to enable comparative evaluation of the relative merits of malaria control measures, and specifically with respect to the design of malaria vaccine trials. The need for standardization of clinical trial design, conduct, analysis and reporting has been also affirmed as a priority area by the Malaria Vaccine Technology Roadmap.

## Background

The development and deployment of new and improved intervention methods for malaria control shows promising signs of reducing significantly the global burden of malaria. However, the search for more effective control methods still has very high priority. Controlled trials remain essential for the rigorous assessment of the potential impact of new tools and strategies to reduce morbidity and mortality caused by malaria. In recent years, there have been a considerable number of randomized controlled trials of new malaria interventions directed at children, including trials evaluating candidate malaria vaccines, insecticide-treated bed nets (ITN) and intermittent presumptive or preventive therapy in infants (IPTi). The appropriate choices of the primary end-points in such trials and the measurement methods are prerequisites for the proper evaluation of the interventions. The end-points and measurement methods must allow comparability of the performance of the same intervention in different locations and age groups and over time at the same location. In addition, the comparability of performance of alternate control measures, or combinations of measures, relies on standardized methods of assessment.

To prepare for a World Health Organization (WHO) consultation on design issues in malaria vaccine trials, the methods that have been used in reported malaria preventive intervention trials to estimate efficacy against clinical malaria and related end-points were reviewed and the strengths and weaknesses of the different approaches were summarized. The aim is to provide a resource for those planning clinical trials of preventive malaria interventions. The target audience is clinical trialists, statisticians and other technical personnel. A companion paper, resulting from a WHO consultation on the issues, was directed at policy makers, funders and regulators [1].

## Methods

### Identification of studies

Reports of trials published between 1990 and 2007 evaluating the impact of preventive malaria interventions were identified (specifically vaccines, impregnated bed nets and IPTi). Trial reports were found primarily through PubMed database searches. Papers were sought using the following search terms: malaria vaccin*, malaria vaccines [MeSH], (malaria or insecticide-treated or impregnated or pyreth* or deltamethr*) and (bednet or bed net or mosquito net or curtain), malaria ipti, malaria intermittent therapy infants, malaria presumptive therapy infants, malaria preventive therapy infants, malaria preventive clinical trial. Further trials were identified through the references of retrieved articles. In addition, investigators responsible for the design and analysis of clinical trials of preventive interventions against malaria were interviewed as part of a WHO consultation process on design of phase 3 trials of malaria vaccines. Each interviewee was asked to identify further eligible studies. Data on trial methods and reporting for each study were extracted into tables and are summarized [see additional file 1].

### Eligibility criteria

Papers were included if they reported randomized controlled trials of preventive malaria interventions with a primary or secondary objective stated as the estimation of efficacy against clinical malaria, severe malaria or all-cause mortality. Trials were excluded, to coincide with the intended scope of the review, if they were conducted in adult populations or in areas of unstable malaria transmission (i.e. annual entomological inoculation rate < 1 or little evidence of acquisition of clinical immunity in the population) or they did not report incidence end-points.

## Results

29 randomized clinical trials of malaria preventive interventions in children published between 1990 and 2007 were identified, which reported estimation of efficacy against clinical malaria, severe malaria or all-cause mortality. Six trials, all of insecticide-treated bed nets or curtains, were cluster randomized. The other 23 were individually randomized.

### Choice of the primary end-point

The trials could be classified broadly into two groups. The largest group consisted of trials designed primarily to detect an effect on clinical malaria. In some of these both clinical malaria and anaemia are described as efficacy measures in the same trial. In several trials, it was not possible to identify whether a single primary end-point had been specified amongst several end-points that were ostensibly reported as "co-primaries".

The other group of trials, all of ITNs, were those designed to detect an effect on all-cause mortality. Severe malaria was a primary outcome measure in only one trial [2].

### Clinical malaria as a primary end-point

All malaria vaccine trials [3-8] and several trials of IPTi [9-15], whose stated main objective was the estimation of efficacy against clinical malaria, had as their primary end-point the incidence rate of the first episode of malaria (time to first or only episode of malaria). For this end-point, following entry into the trial, only the first episode of clinical malaria for each child contributes towards the calculation of the malaria incidence rate. Episodes of the disease after the first are ignored. Each child contributes a variable amount of time at risk to the denominator for the rate calculation, from the beginning of the efficacy follow-up period (i.e. from first vaccination for "intention to treat" analyses and generally 14 days from final vaccination for "according to protocol" analyses) until the onset of the first episode of malaria or the end of the follow-up period (whichever is the shorter). The point estimate of efficacy is calculated as 1-(incidence rate ratio) or by using Cox proportional hazards regression models or Poisson regression models, if adjustment for other factors is necessary. The use of such "time-to-first-event analyses" has been common in evaluating preventive interventions against malaria, unlike in some studies of other infectious diseases [16-19]. Methods such as these that allow variable follow-up time between individuals to be taken into account are generally accepted to be preferable for field efficacy trials. A simple comparison of the ratio of proportions infected remains the method of choice for artificial challenge trials; here equal follow-up time for each individual is a reasonable assumption. However, the utility of "time-to-first-event analyses" for public health policymakers has been questioned as it is argued that it does not measure the overall burden of malaria, which includes second and subsequent episodes in the same individual.

An alternative end-point for trials designed to examine the overall impact against clinical malaria is the rate of all episodes of malaria. Only one trial was identified reporting multiple episodes of malaria as its primary end-point [20]. For this end-point, the total number of malaria episodes are counted, including multiple episodes in the same child, using a rule for the minimum number of days between episodes to distinguish a "new" episode from a continuation of the previous one (usually 28 days but dependent on the chemotherapy used). In some trials molecular markers have also been used to distinguish recurrences from new infections. This end-point may be of more relevance to public health than one based upon the first or only episode of clinical malaria. However, the analysis of data including multiple episodes in the same child is not straightforward as multiple episodes are not independent events. That is, once a child has had one episode, the child is more likely to have another episode, for a variety of reasons, than a child who has never had an episode. Thus, attacks of malaria tend to "cluster" within individuals. Complex models are needed to analyse these sorts of data, on which there is still not a consensus and on which further methodological research is required. To date, unease about this issue seems to have inhibited the use of this as a primary end-point in most trials. A recent WHO consultation highlighted the need for an explanatory document, outlining the merits and disadvantages of the different approaches to measurement of efficacy against clinical malaria in preventive intervention trials[21]. Drafting of such a document is underway.

#### What is clinical malaria?

Defining what constitutes an episode of clinical malaria is not straightforward in areas in which the disease is highly endemic. In such areas, at any one time, a significant proportion of children in a community may have malaria parasites in their blood. Many will be asymptomatic and others will have symptoms consistent with malaria. In some of this latter group the symptoms may be directly attributable to malaria but, because malaria symptoms are not specific to the illness, in others the symptoms may be due to another condition and the malaria parasites in their blood are merely coincidental. In general, the higher the blood parasite load, the more likely it is that the symptoms are due to malaria. Thus, in preventive trials, it is common practice to define clinical malaria as being present in those who have symptoms and signs consistent with malaria and who also have a density of parasites in their blood greater than some specified level. Choice of the appropriate minimal parasite density level (cut-off) for defining malaria will depend upon the endemicity of malaria in the study area. For example, in areas where malaria is relatively uncommon, the finding of any malaria parasites in the blood of someone presenting to a health facility with fever provides a sensitive and specific diagnosis of the disease. For malaria endemic areas, Smith *et al *[22] devised a method for calculating the sensitivities, specificities and malaria attributable fraction of different parasite density cut-offs, using baseline data on the prevalence of different levels of parasite density measured in children in the community not presenting with malaria symptoms. To choose the appropriate cut-off level in a specific intervention trial, it is important that the data used for deriving this level are from the same epidemiological setting as the intervention trial (including age, transmission intensity, health care facilities and interaction with study staff). This is often not specified in the reports of trials.

In recent malaria vaccine trials in children, efficacy estimates using different parasite density cut-offs, in the same trial, have been presented. Case definitions with lower specificity (lower parasite cut-off levels) should theoretically yield lower point estimates of efficacy. Curiously, this expected effect has not been seen generally in the trials reviewed. Further research into this issue is warranted. Also, it would aid interpretation if the sensitivities and specificities of the various case definitions and parasite density cut-offs used, as derived by the Smith *et al *method, were presented in publications of intervention trials. This has generally not been done.

A different approach to defining clinical malaria was used in an ITN trial performed in Côte d'Ivoire [23]. In this trial, each participant was regularly tested for the level of malaria parasites in their blood. Each child was then assigned a probability of having suffered an episode of clinical malaria in a given period, according to the highest parasite density recorded during that period, the probability being determined using a logistic regression approach. The sum of the probabilities across individuals was taken as the total "episodes" of malaria in the group. For analysis purposes, only one episode was included within any six-week period. While this approach does not go as far as avoiding any estimation of incidence of malaria at the individual level (as has been proposed as a possibility[24,25]), this study does move away from classifying outcomes in an individual child in a simple binary way.

Another measure of clinical malaria was used in a trial of intermittent preventive therapy with sulphadoxine/pyrimethamine (SP) and iron supplementation performed in Kenya[26]. The analysis was based on the risk rather than the rate of malaria. Thus, the number of cases of malaria was divided by the number of children at risk rather than by the person-time at risk.

#### Use of pre-treatment

In trials of malaria vaccines, an assessment has commonly been made of the effect of the vaccine on the incidence of parasitaemia as well as on clinical malaria. The former has been measured by taking repeated blood smears from some children following vaccination. In order to be able to measure new infections, in most trials, anti-malarial treatment has been given prior to vaccination to clear asexual parasitaemia. Thus, in five out of eight malaria vaccine efficacy trials in children, participants were treated for malaria at the start of the trial. A sixth trial used amodiaquine/SP pre-treatment in a separate cohort, where only anti-infection efficacy data was generated[7]. In one trial, there was a double randomization (by vaccine vs control and pre-treatment vs no pre-treatment) to examine the impact of pre-treatment on vaccine efficacy[27]. Although the numbers were small in this study, and the primary end-point was parasite density, there was a marked "sterilizing" effect of SP pre-treatment for several weeks into the follow-up period, such that efficacy could not be determined in the pre-treatment group. Epidemiological studies in Kisumu (unpublished) and Mali[28] have been conducted in children to estimate the duration of effect of artemether/lumefantrine and SP, respectively, on time to clinical malaria. In the latter study, SP pre-treatment delayed the median time to first clinical episode from 38 days to 68 days. The data now available suggest that pre-treatment of vaccine trial participants may have complex effects of unpredictable duration on observed vaccine efficacy. Thus, it may be appropriate to restrict pre-treatment to trials where time to infection is the primary end-point. Where pre-treatment is used, it may be a major complicating factor for interpretation of morbidity end-points.

#### Surveillance methods

All malaria vaccine trials in children published to date have included some form of active case detection (ACD) for at least some trial participants. Two trials [3,7] used active surveillance in one group and passive case detection (PCD) only in other group(s) in the same study. In one of the study cohorts in the study of Alonso *et al *[7] the scheduled interaction between study staff and participants during the efficacy follow-up period was less than for any other cohort in the vaccine studies identified. Participants were visited at home once a month to establish presence in the study area and to record any unreported serious adverse events but the visits did not involve malaria morbidity surveillance. Clinical malaria episodes, identified only by children presenting at a clinic, are therefore likely to have been more severe, on average, than those experienced in other vaccine trials in which active case detection was employed. The clinical malaria cases identified in this study are likely to accord more closely to those encountered in normal health practice in the study area and, therefore, it might be argued that the trial results will be more relevant to public health.

The criteria used to trigger the taking of a blood smear to assess for a possible clinical malaria episode varied from trial to trial. This also affects interpretation of efficacy results. Thus, the results of trials must be interpreted in the light of the case detection systems used.

In some trials, both ACD and PCD were used to detect clinical malaria cases and efficacy results were reported for cases detected by each of these methods[3]. With such dual surveillance the severity of the clinical malaria detected by PCD is likely to be less severe, on average, than if there had been no ACD. In trials where the choice of the end-point is designed to approximate what might happen in the usual health care system the use of ACD should be avoided. Monthly visits to trial participants for purely safety information would appear to be an appropriate level of interaction for studies planning PCD only surveillance.

#### Blood smear methods

There are three methods for calculating parasite density that have been employed in published intervention trials. Firstly, reading 100 or 200 high power fields, with the assumption that the volume of each high power field is 1/500 μl. Secondly, an assumption that the total white cell count is 8,000/μl and reading to 200 white blood cells. Thirdly, a calculation of white cell count individually and then reading to 200 WBC, with adjustment using the calculated count. It is difficult to compare the results of studies in which different counting methods have been used. Standardization on one method is desirable for accuracy, precision and to aid comparisons of the results in different trials and is essential for a multi-site licensure trial. Double reading of all blood smears for efficacy end-points is also undertaken, and is highly desirable, in most intervention trials. The elements of slide taking and reading that require standardization include: staining of blood smears including where Field's stain and Giemsa stain are used; procedure for double and third reading of smears; internal quality control (QC) procedures; external QC of a proportion of blood smears and the process for resolution of discrepancies found through external QC.

### Other end-points

While clinical malaria has been the primary end-point in most studies of preventive interventions studies, some studies have measured the impact on more severe disease or death and others have evaluated the impact on hospital utilization for any cause.

#### Severe malaria, malaria-related mortality and all-cause mortality

Whilst the presenting features and, to some extent, predictors of mortality in hospitalized cases of malaria have been adequately described in a handful of settings in sub-Saharan Africa [29-40], only a single published preventive intervention trial included severe malaria as the primary end-point[2], though other trials have had this as a secondary end-point. Identifying deaths due to malaria is problematic in situations where post-mortem is uncommon and many children may die out of a hospital setting. There is a well established precedent for the use of verbal autopsies to assign causes of death in both sub-Saharan Africa[41] and Asia[42], but there is limited experience of using these methods to assess the impact of a preventative intervention on malaria-related mortality[43] and the sensitivity and specificity of the assignment of a malaria death is poor. However, with a good surveillance system all deaths from any cause can be identified with considerable reliability. Thus, in some trials, specifically some of the ITN trials [2,43-46], death from any cause has been the primary end-point. Whilst not appropriate for a licensure trial, the impact of a vaccine on this end-point may be important for assessing the public health impact of a malaria vaccine post-licensure, and might be a focus for Phase 4 studies. In some settings, an effect on severe malaria may also be ascertainable in a study in which all-cause mortality is the primary end-point[2].

#### Hospital admissions

In some trials an attempt has been made to estimate the extent to which a preventive malaria intervention has reduced the overall burden of illness on the health service, such as the impact on all-cause clinic or hospital attendance. For example, this measure was included in the evaluation of the Asembo/Gem Kenyan ITN trial [47]. Over 20,000 clinic attendances were included in the primary analysis and a statistically significant efficacy of 27% against all-cause clinic attendance was reported. No sample size calculation was provided, but it appears likely that such large numbers are necessary to provide adequate power to detect such an effect. This end-point, though potentially difficult to measure in some circumstances, would be highly informative for those making implementation decisions in malaria control, as it provides a general measure of the potential saving to the health service.

#### Use of multiple efficacy endpoints

A relatively large number of possible efficacy end-points are potentially available to malaria investigators and malaria preventive intervention trials may be particularly vulnerable to the multiple comparisons problems in trial design. Many of the trials reviewed reported a large number of different efficacy end-points (more than 20 in some trials). Furthermore, it is not generally made clear in papers how many efficacy end-points were analysed but were not reported. A ranking of end-points pre-unblinding, in the analytic plan, with publication in the pre-specified rank order would be one way to address concerns about multiple comparisons. Conservative methods such as adjusting significance levels for the number of end-points examined have not been used in trials reported to date. In practice, in addition to the primary end-point (if one is specified!) the sum of the evidence from all of the end-points measured is usually taken into account, together with their biological plausibility and their consistency, in the overall assessment of the potential impact of an intervention. Assessment would be aided if, for all trials, the reporting and analysis plans were made available for review together with the trial protocol.

## Conclusion

There is a wealth of data available from randomized controlled trials of malaria preventive interventions directed at children, much published in recent years. Further information will become available through ongoing iPTi trials and the planned large multi-site phase 3 trial of the GlaxoSmithKline/Malaria Vaccine Initiative vaccine candidate RTS, S. Attention to the design issues identified above will aid the generation of data that can inform public health decisions.

It is notable that many of the trials identified in this review report the incidence rate of first episodes of clinical malaria as the primary endpoint. Given the public health interest in a potential new intervention's impact on the community burden of clinical malaria, further attention to analysis methods for the incidence rate of all episodes of malaria appears to be one priority for those undertaking clinical trials in this field. This may help bridge the gap between proof-of-concept and evaluation of public health benefit for preventive interventions against clinical malaria.

An ambitious, but highly laudable goal, stated as a priority area in the strategic framework of the Malaria Vaccine Technology Roadmap[48], is clinical trial standardization. The hope is that those engaged in preventive intervention trials against malaria, and especially those engaged in vaccine trials, will discuss and come to agreement on optimal trial design, analysis method and reporting requirements. Having agreed these aspects the plan is to produce standardized clinical trial methods, protocols and reporting templates. This report forms one part of the background to such harmonization and standardization efforts. The Initiative for Vaccine Research, World Health Organization is engaged in ongoing activities to facilitate norms, standards and best practice in this area[21].

## Competing interests

The authors declare that they have no competing interests.

## Authors' contributions

VSM performed the literature search, extracted the data on methods and reporting and wrote the first draft of the manuscript. ZR conceived the scope for the review and provided input into the final manuscript. PS provided input and review into the final manuscript.

## Supplementary Material

Additional file 1 **Tables 1–4**. Tables summarising methods and reporting for identified randomized controlled trials of preventive malaria interventions. Click here for file
