# Supplementary material for: Clinical trials to estimate the efficacy of preventive interventions against malaria in paediatric populations: a methodological review
Source: Malar J. 2009 Feb 10;8:23. doi: 10.1186/1475-2875-8-23 (PMC2646744; doi:10.1186/1475-2875-8-23)
Supplement: Additional file 1 — Tables 1–4. Tables summarising methods and reporting for identified randomized controlled trials of preventive malaria interventions. [file 1475-2875-8-23-S1.doc]

**Table 1 Malaria vaccine trials in children**

| Intervention [Ref] | **Site (Year)** | **Number enrolled (age)** | **Primary end-point** | **Pre-Rx** | **Surveillance** | **Smear obtained** | **Case Definition** | **Reported Efficacy** | **EV** | **Sens/Spec** | **Smear method** |
| --- | --- | --- | --- | --- | --- | --- | --- | --- | --- | --- | --- |
| SPf66 at 1-5 yr[3] | Ifakara 1994 | 631 (mean 3.4 yr) | TFOEM | Y | PCD in 2 groups. Weekly ACD in 1 group. Multiple CSS. | Clinic or FW if fever or 24 hr Hx fever | Fever, >20,000 ppm by PCD | 31% (to 44 weeks after dose 3) | N | 83%/82% - PCD cases in trial | Per 200 WBCs with assumed ct |
| SPf66 at 1,2,7 mo[4] | Ifakara 1999 | 1207 (1 mo) | TFOEM | N | PCD with CSS at 8,12,24 mo | Clinic if fever or 24 hr Hx fever | Fever, >0 ppm by PCD | 2.0% (to age 24 months) | N | 100%/88% - - prev data | Per 200 WBCs with assumed ct |
| SPf66 at 2-15yr[5] | Thai-Burma border 1996 | 1348 (1/3 <6yr) | TFOEM | N | ACD daily FW visits + PCD at 1 unit clinic and 1 MSF hospital | Clinic if fever or symptom(s) | >=38.1C or symptoms, >0 ppm by ACD/PCD | -9% (to 12 mo after dose 3) | n/a | Not given | Calculated cell counts |
| SPf66 at 6-11mo[6] | URD, The Gambia 1995 | 669 (mean 8.4 mo) | TFOEM | Y | ACD twice weekly visits, PCD at 6 health centres & 1 clinic | Clinic or FW if fever | Fever, >6,000 ppm by PCD & ACD | 8% over 15 weeks | N | 86%/86% | 1ppHPF=  500ppm |
| Combo B at 5-9 yr[27] | South Wosera District, PNG 2002 | 120 (mean 7.5 yr) | Geometric mean parasite density^*^ | ½ Y, ½ N | Weekly ACD with PCD at 1 clinic. 4 weekly CSS | Clinic or FW if fever or 72 hr Hx fever | Fever or 72 hr Hx fever >8,000 ppm ACD/PCD | <0% - figure not given | N | Ref given | Per 200 WBCs with calculated ct |
| RTSS at 1-4 yr[7] | Manhica, Mozambique 2004 cohort 1 | 1605 (mean 35 mo) | TFOEM | N | PCD at 1 clinic with CSS at 8.5, 21 mo | Clinic if fever or 24 hr Hx fever | Fever, >2,500 ppm | 29.9% (to 6 mo after dose 3) | N | 95%/91% - prev data | Per 200 WBCs with assumed ct |
| RTSS at 10,14,18 weeks[49] | Manhica, Mozambique | 214 (10 weeks) | Safety.  TFOEM secondary | Y | 2 weekly active detection of infection & PCD | Clinic if fever or Hx fever or pallor | Fever,  >500 ppm | 65.8% (to 6mo after dose 3) | N | >90% | Per 200 WBCs with assumed ct |
| FP9/MVA ME-TRAP 12-84 mo[8] | Kilifi, Kenya 2006 | 405 (mean not stated) | TFOEM | Y | Weekly ACD with resident FW | FW if documented fever only | Fever, >2500 ppm ACD | <0% (to 9mo after dose 3) | N | Ref given | Per 200 WBCs with calculated ct |

TFOEM=time to first or only episode of malaria Pre-Rx=Pre-treatment EV=efficacy variation with alternate case definitions

Sens/Spec = Sensitivity and specificity of case definition ppm=parasites per μlitre

ACD, PCD = active or passive case detection CSS = cross sectional survey AQ = amodiaquine SP=Sulphadoxine/pyrimethamine

AS= artesunate FW= field worker URD= upper river division

KND= Kassena Nankana District ^*^ - subjects with zero values excluded from analysis

**Table 2 Trials of IPTi**

| Intervention [Ref] | **Site (Year)** | **Number enrolled (age)** | **Primary end-point** | **Surveillance** | **Smear obtained** | **Case Definition** | **Reported Efficacy** | **EV** | **Sens/Spec** | **Smear method** |
| --- | --- | --- | --- | --- | --- | --- | --- | --- | --- | --- |
| SP IPTi at 2,3,9 mo[10] | Ifakara 2001 | 701 (2 mo) | TFOEM | PCD at 1 clinic with single CSS at 12 mo | Clinic if fever or 24 hr Hx fever or appeared pale | Fever, >0 ppm | 59% (to age 12 mo) | N | 100%/97% - prev data | Per 200 WBCs with assumed ct |
| AQ IPTi or Fe at 3,5,7 mo[11] | Muheza, Tanzania 2003 | 291 (12-16 weeks) | TFOEM | PCD at 3 health centres | At all clinic attendances | Fever or 72 hr Hx fever, >0 ppm | 63.4% over 6 mo (2 mo from last dose) | N | Not given | Per 200 WBCs with assumed ct |
| IPT with SP in 2-36mo[26] | Asembo 2004 | 546 (mean 11 mo) | Hb & clinical malaria | Daily FW, 2 weekly ACD, 4 weekly CSS. PCD | Not by FW. For PCD not stated. | Fever, >0 ppm  ACD+PCD | 53% over 12 weeks (p=0.07) | n/a | Provided – not used | Per 300 WBCs, assumed ct |
| AS+SP IPTi at 2 - 59 mo[12] | Niakhar, Senegal 2006 | 1088 (2-59 mo) | TFOEM | Weekly ACD + PCD at 3 health centres | Clinic or FW if fever or 24 hr Hx fever or vomiting | Fever or 24 hr Hx fever or vomiting, >3,000 ppm  ACD&PCD | 86% over 13 weeks | N | Not given | Per 200 WBCs with assumed ct |
| AS+SP IPTi at 2-59 mo[20] | KND, Northern Ghana 2006 | 2485 (2 mo) | Time to PCV<24%, (all malaria) | PCD at 11 health centres and 1 hospital clinic | Health centre/ clinic if fever or 48 hr Hx fever | Fever or 48 hr Hx fever with >5,000 ppm | 16.3% for all malaria episodes to age 24 mo | N | Not given | Not stated |
| SP at 3,4,9 mo[13] | Manhica,  Mozambique 2006 | 1503 (3 mo) | TFOEM | PCD at 1 clinic + CSS at 12 and 24 mo | Clinic if if fever or Hx fever or pallor | Fever with >0 ppm | 22.2% to age 12mo | 26.4% for most specific case definition | 100%/84% | Per 200 WBCs with assumed ct |
| SP at 3,9,15 mo[14] | Northern Ghana 2007 | 1200 (3 mo) | TFOEM & all episodes | PCD at 1 clinic + CSS 6 monthly | Clinic if fever or Hx fever | Fever or 48 hr Hx fever with >0 ppm | 14.7% TFOEM to age 24 mo.  22% all episodes | N | Not given | Not stated |
| SP at 3,9,15 mo[15] | Ghana 2007 | 1070 (3 mo) | TFOEM | Monthly CSS | At all FW visits | Fever or 48 hr Hx fever with >500 ppm | 18.4% for first episode  20.3% for all episodes | N | >90% | Per 200 WBCs with assumed ct |
| SP at 3,9,15 mo[50] | Gabon 2007 | 1189 (3 mo) | Time to first episode of anaemia | PCD & monthly ACD | Clinic or FW if fever or Hx fever | Fever or 48 hr Hx fever | 22% to age 18mo | N | Not given | Known volume of blood |

Key to Table 2:

TFOEM=time to first or only episode of malaria

Pre-Rx=Pre-treatment

EV=efficacy variation with alternate case definitions

Sens/Spec = Sensitivity and specificity of case definition

ppm=parasites per μlitre

ACD,PCD active or passive case detection

CSS = cross sectional survey

AQ = amodiaquine

SP=Sulphadoxine/pyrimethamine

AS= artesunate

FW= field worker

URD= upper river division

KND= Kassena Nankana District

| Intervention [ref] | **Site (Year)** | **Number enrolled (age)** | **Primary end-point** | **PreRx** | **Surveillance** | **Smear obtained** | **Case Definition** | **Reported Efficacy** | **EV** | **Sens/Spec** | **Smear method** |
| --- | --- | --- | --- | --- | --- | --- | --- | --- | --- | --- | --- |
| D-P/Fe in 2-11mo[9] | Ifakara 1997 | 832 (age 8 weeks) | TFOEM anaemia | - | PCD. 3 CSS at 5,8,11 mo | Clinic if fever or 24 Hx fever | Fever, >0 ppm. PCD | 60% &57% over 12 mo | n/a | 100%/88% | Per 200 WBCs, assumed ct |
| D-P in 1-9 yr[51] | Muheza 1997 | 249 (1-9 yr) | Multiple malaria | - | Weekly ACD only. | FW if fever or 48 hr Hx fever | Fever, >5000ppm. ACD | 39% & 42 % (for 1-4 & 5-9yr) | N | Not given | Per 200 WBCs, assumed ct |
| Vit A in 6-60 mo, 5 doses[52] | North Wosera, PNG 1999 | 480 (mean not stated) | Multiple malaria | N | Weekly ACD. PCD at 1 health centre. | Clinic or FW if fever or 72 hr Hx fever | Fever or 72 hr Hx fever >8000ppm§  ACD+PCD | 30% over 13 mo ACD/PCD | N | 76%/80% | Per 200 WBCs, calculated ct |

**Table 3 Malaria prophylaxis and Vitamin A trials**

Key to table 3:

D-P Dapsone/Pyrimethamine

TFOEM = Time to first or only episode of malaria

§ Paper does not state that children were considered not at risk for a period after onset of each episode

EV = Efficacy variation with alternate case definitions

ACD,PCD active or passive case detection

| Intervention [ref] | **Site (Year)** | **Number enrolled (age)** | **Primary end-point** | **PreRx** | **Surveillance** | **Smear obtained** | **Case Definition** | **Reported Efficacy** | **EV** | **Sens/Spec** | **Smear method** |
| --- | --- | --- | --- | --- | --- | --- | --- | --- | --- | --- | --- |
| ITNs in 1-9 yr[44] | 5 areas in The Gambia 1995 | 18911 (birth-9 yrs) | All-cause mortality | N | Annual DSS & CSS. Weekly ACD | At CSS | Confirmed death | 25% over 1 yr | - | - | - |
| ITNs in 1-59 mo[2] | Kilifi, Kenya 1996 | 22998 | All-cause mortality | N | 6 monthly DSS | In hospital – not stated | Confirmed death | 30% over 2 years | - | - | - |
| ITNs in 6-59 mo[43] | KND, North Ghana 1996 | 32335 child years | All-cause mortality | N | 6 monthly DSS | - | Confirmed death | 17% over 2 years | - | - | - |
| ITCs in 6-59 mo[45] | Burkina Faso 1997 | 29282 child years | All-cause mortality | N | Annual census + 3 weekly ACD | - | Confirmed Death | 15% over 2 yrs | - | - | - |
| ITNs in 0-59 mo[46] | Asembo/Gem Kenya 2003 | 221 villages, 35932 child-years | All-cause mortality | N | 6 monthly DSS | - | Confirmed death | 16% over 2 years | - | - | - |
| ITNs in 1-24 mo[53] | Asembo Bay Cohort 2003 | 860 infants (day 28) | Not clear | N | 2 weekly ACD.4 weekly CSS. PCD. DSS. | Not by FW. For PCD not stated. | Fever, >0 ppm  ACD+PCD | 52% age 1-24 mo | N | Provided by age group – not used | Per 300 WBCs, assumed ct |
| ITNs in 0-59 mo[47] | Asembo Kenya 2003 | 79 villages, 20915 clinic visits | All-cause clinic visits | N | PCD at 13 health centres. 6 monthly DSS | Not stated | Assigned by clinic staff PCD | 27% ITN-associated reduction | N | (30% drop malaria visits) | - |
| ITNs in 0-59 mo[23] | Northern Côte d’Ivoire 2005 | 426 (mean 27 mo) | Clinical Malaria** | N | Daily ACD for 7 days every 6 weeks | Nurse if fever, Hx fever or symptoms | Sum of probabilities** ACD | 56% over 12 months | n/a | - | Per 200 WBCs, assumed ct |
| ITNs in 0-59mo[54] | NW Burkina Faso 2006 | 3387 (from birth) | Mortality & multiple malaria | N | Twice weekly ACD. 6 monthly CSS. | By FW if fever or 48 hr Hx fever | Fever, >5,000ppm | RR 3.11 for 0-5mo over 26 mo | Y | Not given | Per 200 WBCs, assumed ct |

**Table 4** **ITN trials**

Key to Table 4:

ITN – insecticide treated net

ITC – Insecticide treated curtain

DSS demographic surveillance system (frequency of surveys)

ACD – active case detection (for deaths in this context)

KND- Kassena Nankana District

** Denominator child-days at risk for periods of ACD only. Numerator: for each febrile parasitaemia, probability due to malaria estimated. Only 1 episode per 7 days considered. Total number of episodes for each child estimated from sum of probabilities over efficacy follow-up.
